# Preoperative hypomagnesemia as a possible predictive factor for postoperative increase of transvalvular pressure gradient in hemodialysis patients treated with transcatheter aortic valve implantation

**DOI:** 10.1080/0886022X.2022.2094272

**Published:** 2022-07-07

**Authors:** Satoshi Masuyama, Masayuki Mizui, Koichi Maeda, Kazuo Shimamura, Yusuke Sakaguchi, Masashi Morita, Toru Kuratani, Isamu Mizote, Daisuke Nakamura, Yasushi Sakata, Yoshiki Sawa, Shigeru Miyagawa, Yoshitaka Isaka

**Affiliations:** a Department of Nephrology, Osaka University Graduate School of Medicine, Suita, Japan; b Department of Cardiovascular Surgery, Osaka University Graduate School of Medicine, Suita, Japan; c Department of Minimally Invasive Cardiovascular Medicine, Osaka University Graduate School of Medicine, Suita, Japan; d Department of Inter-Organ Communication Research in Kidney Diseases, Osaka University Graduate School of Medicine, Suita, Japan; e Department of Cardiovascular Surgery, Sakurabashi Watanabe Hospital, Osaka, Japan; f Department of Cardiovascular Medicine, Osaka University Graduate School of Medicine, Suita, Japan; g Department of Cardiovascular Surgery, Osaka Police Hospital, Osaka, Japan

**Keywords:** Magnesium, TAVI, valve dysfunction, hemodialysis

## Abstract

**Background:**

Patients undergoing maintenance hemodialysis (HD) with severe aortic stenosis are at a high risk for bioprosthetic valve dysfunction after transcatheter aortic valve implantation (TAVI). Currently, preoperative factors that predict the occurrence of valve dysfunction after TAVI on HD patients remain to be elucidated. The aim of this study is to analyze the association between preoperative clinical factors and valve stenosis after TAVI on HD patients.

**Methods:**

Twenty-four of HD patients who underwent TAVI at our institution between April 2012 and January 2016 were analyzed. The mean aortic transvalvular pressure gradient (MPG) and effective orifice area index (EOAi) were assessed by serial echocardiography. Associations between preoperative clinical factors and time-series changes in MPG were examined using mixed-effects linear regression model for repeated measures.

**Results:**

Three patients developed severe structural valve deterioration with calcific valve stenosis requiring reoperation. A multivariate linear mixed-effects model showed that lower serum magnesium (sMg) levels were associated with the increase of MPG after TAVI (beta-coefficient = 0.019, *p* = 0.03). No correlation was observed with serum calcium, phosphorus, or intact parathyroid hormone. Time-series changes of MPG and EOAi had significant difference between lower and higher sMg group. All 3 of the patients who underwent reoperation showed lower preoperative sMgs.

**Conclusion:**

Among bone-mineral metabolism markers, preoperative hypomagnesemia was associated with the increase of MPG after TAVI, suggesting that hypomagnesemia could predict post-TAVI valve dysfunction in HD patients. Further studies with larger sample sizes are warranted.

## Introduction

Transcatheter aortic valve implantation/replacement (TAVI/TAVR) is a useful alternative to surgical aortic valve replacement (SAVR) in severe aortic stenosis (AS) [1,2], especially for surgical high-risk patients [3–6]. Bioprosthetic valve could gradually degrade and induce dysfunction which is classified into structural valve deterioration (SVD) or not. SVD mainly presents as leaflet calcification resulting in valve stenosis, but also as tear, stent fracture and creep resulting in regurgitation [7]. Additionally, TAVI-related factors such as crimpling, balloon expansion, asymmetric expansion and residual native annular calcification are also causes of valve dysfunction. Although the incidence of SVD in 5 years after TAVI is equivalent to that after SAVR, much higher incidence of SVD in 10 years was reported around 30% after TAVI, as compared with <10% after SAVR [8,9]. Therefore, long-term durability of implanted valve is still under controversy [10].

Advanced chronic kidney diseases could be a further risk factor for SVD due to valve stenosis [7,11]. Since calcific AS is prevalent in patients on maintenance hemodialysis (HD) [12], it is conceivable that durability of bioprosthetic valve emerges as a problem in HD patients. Indeed, increased rate of valve failure was recently reported in HD patients as compared in non-HD patients during 2 years after TAVI [13]. Five out of 83 HD patients underwent calcific degeneration. We also have previously reported mid- to long-term outcomes of TAVI in HD patients, and 3 of 25 patients underwent reoperation (transcatheter AVR in transcatheter aortic valve: TAV-in-TAV) due to severe bioprosthetic valve stenosis [14].

Calcification of the aortic valve may be derived from the bone and mineral metabolism such as calcium, phosphorus, and magnesium, which is highly disturbed in HD patients. Currently, predictors of postoperative valve dysfunction after TAVI in HD patients are not fully understood. In this study, we analyzed preoperative clinical factors associating with valve stenosis after TAVI by time-series changes of mean aortic transvalvular pressure gradient (MPG), using mixed-effects linear regression model for repeated measures.

## Materials and methods

### Patients

Twenty-five of HD patients with severe AS underwent TAVI at our institution between April 2012 and January 2016 [14]. Severe AS was defined as aortic valve area (AVA) <0.8 cm^2^, an effective orifice area index (EOAi) <0.5 cm^2^/m^2^, mean pressure gradient ≥40 mmHg, or peak aortic jet velocity ≥4.0 m/s. We included patients with New York Heart Association class ≥ II and STS score ≥10% (otherwise, at least one surgeon and one cardiologist determined that the patient was not suitable for surgery due to comorbidities). In all cases, balloon-expandable bioprosthetic valves made of bovine pericardium were used. The SAPIEN XT (THV 9300) and the SAPIEN (THV 9000) were implanted in 18 (72.0%) and in 7 patients (28.0%), respectively. The transfemoral (TF) and transapical (TA) approaches were performed on 10 patients (40.0%; femoral, 9; iliac, 1) and on 15 patients (60.0%), respectively. Since one patient underwent reoperation due to severe paravalvular leakage soon after TAVI, 24 patients were included in the subsequent analysis.

This study was approved by the institutional review board of Osaka University Hospital. Written informed consent was obtained from all patients.

### Statistical analysis

#### Baseline data

Baseline data were expressed as mean (SD) for normally distributed continuous variables or as median (interquartile range, IQR) for skewed distributed variables, and as number (percent) for categorical variables. Echocardiographic data were prospectively followed until reoperation, death, or last follow-up. AVA and EOA were measured by the continuity equation. EOAi indicates EOA divided by body surface area. To compare baseline levels of serum magnesium (sMg), patients were divided into two groups dependant on bioprosthetic valve conditions after TAVI; re-operation and non-reoperation, non/mild stenosis and moderate/severe stenosis.

#### Mixed-effects linear regression model for repeated measures

A mixed-effects linear regression model was used to analyze the association between preoperative clinical factors and time-series changes in MPG and EOAi, which are one of the most important indices for determining valve stenosis leading to SVD. Next, patients were divided in lower sMg and in higher sMg groups, stratified by two quantiles by median of preoperative sMg. The time-dependant changes in MPG and EOAi were compared between the two groups. Statistical analysis was performed using STATA SE version 16 (Stata Corp LP, College Station, TX, USA).

## Results

### Baseline characteristics

Table 1 shows the demographics including preoperative characteristics and comorbidities. Biological valves were successfully implanted and there were no conversions to SAVR. The mean age was 76.6 years and 58% of the participants were male. A history of cardiac surgery was identified in 9 patients (28%, including 7 with coronary artery bypass grafting), and 5 patients (21%) were diabetic. The median dialysis vintage was 7.5 years (IQR 3.0–17.5 years); the median length of hospital stay after TAVI was 9 days (median, IQR 7.5–11 days); the average follow-up period was 3.4 ± 2.3 years. Serum corrected calcium (Ca), phosphorus (IP) and magnesium (Mg) concentrations showed an average of 9.4 ± 0.7, 4.9 ± 1.0, and 2.4 ± 0.3 mg/dL, respectively, and intact parathyroid hormone (iPTH) 190 pg/mL (median, IQR 130–258 pg/mL). Preoperative echocardiographic data showed AVA of 0.78 ± 0.16 (cm^2^), MPG of 44.7 ± 14.8 mmHg and left ventricular ejection fraction (LVEF) of 60.0 ± 11.9%. Nineteen patients (79%) were taking some types of phosphate binders, 15 patients (63%) were using vitamin D receptor agonists, 7 patients (29%) were using calcium receptor agonists. No patient received Mg supplementation before TAVI. The average dialysate Ca and Mg concentration was 2.81 ± 0.15 and 1.0 mEq/L (1.2 mg/dl).

**Table 1.** Baseline patient characteristics.

	Overall (*n* = 24)
	Median [IQR]/ Average [±SD]/Number [%]
Age, years	76.6 ± 5.1
Male, *n* (%)	14 [58]
BMI, kg/m²	21.4 [18.5–22.5]
BSA, m²	1.49 ± 0.16
HD priod, years	7.5 [3.0–17.5]
Mean blood pressure, mmHg	93 ± 18
Hospitalisation period, days	9 [7.5–11]
Albumin, g/dL	3.6 ± 0.4
Adjusted Calcium, mg/dL	9.4 ± 0.7
Phosphate, mg/dL	4.9 ± 1.0
Magnesium, mg/dL	2.4 ± 0.3
Haemoglobin, g/dL	11.3 ± 1.6
Platelets, ×10^4^/μL	14.5 [12.1–18.0]
PT-INR	1.07 ± 0.09
Triglyceride, mg/dL	94 [77–112]
LDL cholesterol, mg/dL	89 ± 25
Intact parathyroid hormone, pg/mL	190 [130–258]
BNP, pg/mL	669 [471–1102]
Ejection fraction, %	60.0 ± 11.9
Fractional shortening, %	31.8 ± 7.7
Mean aortic valve pressure gradient, mm Hg	44.7 ± 14.8
Left ventricular outflow tract dimension, cm	1.5 ± 2.7
Left ventricular end-systolic volume, mL	51.9 ± 36.8
Left ventricular end-diastolic volume, mL	119.0 ± 46.4
AVA, cm²	0.78 ± 0.16
Ferrous Citrate, *n* (%)	3 [13]
Calcium carbonate, *n* (%)	11 [46]
Lanthenum carboratehydrate, *n* (%)	7 [29]
Sevelamer Bixalomer, *n* (%)	5 [21]
Maxacalcitrioliv Calcitriol i.v., *n* (%)	9 [38]
Alphacalcidol p.o., *n* (%)	8 [33]
Cinacalcet, *n* (%)	7 [29]
Magnesium oxide, *n* (%)	0 [0]
Dialiser membrane area, m²	1.5 ± 0.4
Quantity of blood flow, ml/min	201 ± 21
Dialysate Ca, mEq/L	2.8 ± 0.2
Dialysate Mg, mg/dL	1.21 (all patients)
History of cardiac operation, *n* (%)	9 [38] (CABG: 7)
History of NYHAIV, *n* (%)	6 [25]
History of diabetes, *n* (%)	5 [21]
History of cerebrovascular disease, *n* (%)	14 [58]
History of peripheral vascular disease, *n* (%)	10 [42]
MACCE, *n* (%)	5 [21]
Logistic Euro SCORE	26.6 ± 18.0
STS score	14.9 ± 10.7

Data were expressed as mean (SD) for normally distributed continuous variables or as median (interquartile range) for skewed distributed variables, and as number (percent) for categorical. variables.

BMI: body mass index; BSA: body surface area; HD period: hemodialysis period; AVA: aortic valve area; MACCE: major adverse cardiac or cerebrovascular events; STS: Society of Thoracic Surgeons.

### Magnesium as a factor associated with MPG changes after TAVI

The analysis of the factors that influence the time-series changes in MPG are shown in Tables 2 and 3. In univariate analysis, female gender, higher BMI, lower IP, lower Mg, and higher platelet count, higher LDL-cholesterol level and lower preoperative LVEF were associated with serial increase of MPG after TAVI (Table 2). In particular, sMgs showed a strong correlation with elevation of MPG (beta-coefficient = 0.012 per 1 mg/dL decrease in sMg, *p* < 0.001). After adjustment for these factors and dialysis-related factors and other factors indicated to affect MPG, we found that only sMgs showed a statistical significance in the association with MPG changes after TAVI (Table 3, beta-coefficient = 0.019 per 1 mg/dL decrease in sMg, *p* = 0.03). On the other hand, no significant correlation was found between sMgs and the decrease in EOAi.

**Table 2.** Co-efficiency of baseline factors with time-dependant changes of MPG (univariate mixed-effects linear regression model for repeated measures).

	Coefficient	*p* Value	95% Coefficient interval
Age, years	−0.000081	0.7	−0.00044 to 0.00027
Male, *n* (%)	−0.0069	0.005	−0.012 to −0.0021
BMI, kg/m²	0.00090	0.02	0.00014 to 0.0017
Mean blood pressure, mm Hg	0.000040	0.5	−0.000078 to 0.00016
HD period, years	−0.0017	0.1	−0.00039 to 0.000043
Hospitalisation period, days	0.000042	0.7	−0.00018 to 0.00027
Albumin, g/dL	0.0022	0.6	−0.0054 to 0.0098
Adjusted Calcium, mg/dL	−0.0015	0.3	−0.0041 to 0.0011
Phosphate, mg/dL	−0.0022	0.02	−0.0040 to −0.00040
Magnesium, mg/dL	−0.012	<0.001	−0.017 to −0.0063
Haemoglobin, g/dL	−0.00020	0.8	−0.0018 to 0.0014
Platelets, ×10^4^/μL	0.00084	<0.001	0.00046 to 0.0012
PT-INR	−0.013	0.2	−0.034 to 0.0077
Triglyceride, mg/dL	0.000052	0.07	−0.0000040 to 0.00011
LDL cholesterol, mg/dL	0.00011	0.007	0.000030 to 0.00019
Intact parathyroid hormone, pg/mL	0.000026	0.1	−0.0000061 to 0.000058
BNP, pg/mL	0.00000055	0.8	−0.0000031 to 0.0000042
Ejection fraction, %	−0.00023	0.05	−0.00046 to −0.0000046
Fractional shortening, %	−0.00034	0.06	−0.00069 to 0.000013
Mean aortic valve pressure gradient, mm Hg	−0.00010	0.3	−0.00030 to 0.000099
Left ventricular outflow tract dimension, cm	−0.000034	0.9	−0.0016 to 0.0016
Left ventricular end-systolic volume, mL	0.000065	0.2	−0.000030 to 0.00016
Left ventricular end-diastolic volume, mL	0.000020	0.5	−0.000042 to 0.000082
AVA, cm²/m²	−0.0047	0.5	−0.019 to 0.0097
History of cardiac operation	0.0022	0.4	−0.0027 to 0.0070
History of NYHAIV	0.0010	0.7	−0.0035 to 0.0055
History of diabetes	−0.0011	0.7	−0.0063 to 0.0041
History of cerebrovascular disease	−0.0072	0.004	−0.012 to −0.0023
History of peripheral vascular disease	0.00081	0.8	−0.0047 to 0.0064
MACCE	0.013	0.002	0.0049 to 0.021
Logistic Euro SCORE	0.000094	0.8	−0.00016 to 0.00021
STS score	0.00022	0.8	−0.00039 to 0.00048

All covariates are based on preoperative values. Positive co-efficiency means that MPG increases as the variable is larger.

**Table 3.** Co-efficiency of baseline factors with time-dependent changes of MPG (multivariate mixed-effects linear regression model for repeated measures).

	Coefficient	*p* Value	95%CI
Male	0.00092	0.9	−0.0093 to 0.011
Phosphate, mg/dL	0.0013	0.7	−0.0050 to 0.0076
Magnesium, mg/dL	−0.019	0.03	−0.037 to −0.0015
Platelets, ×10^4^/μL	0.00059	0.1	−0.00016 to 0.0013
LDL cholesterol, mg/dL	−0.000093	0.2	−0.00022 to 0.000036

### Low magnesium and bioprosthetic valve stenosis after TAVI

**Figure 1.**
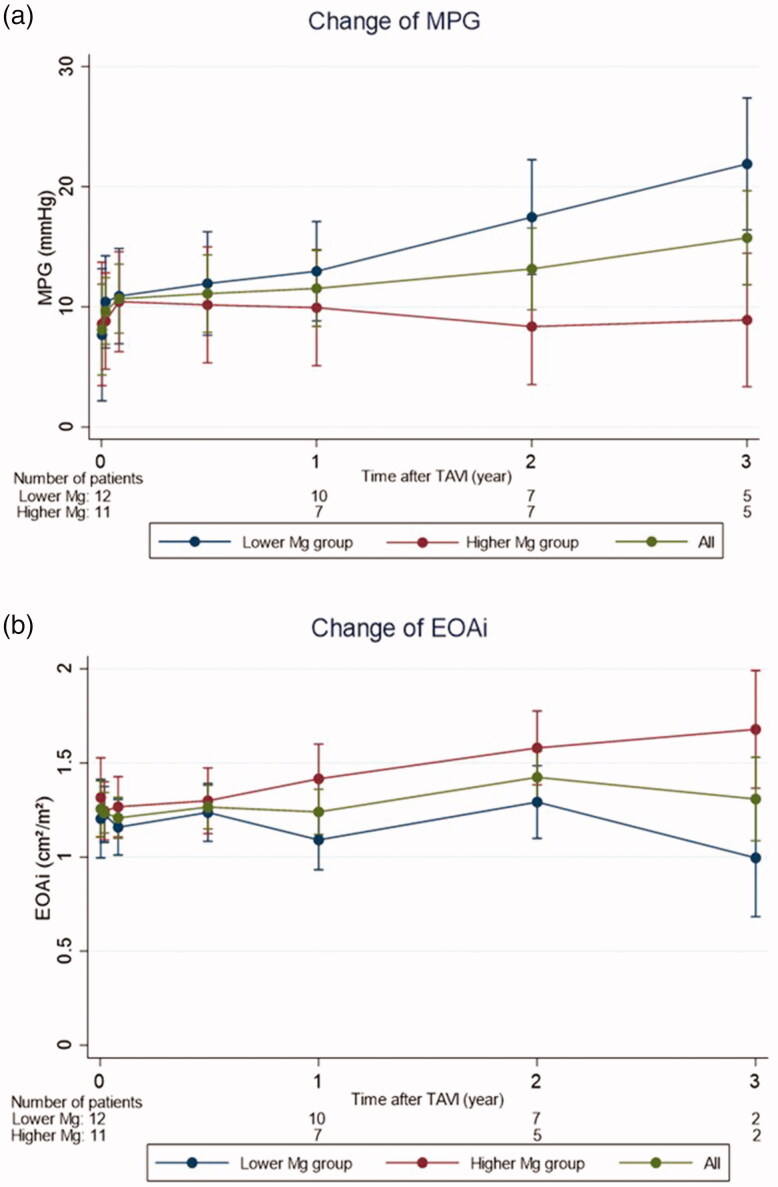
(a) Change of MPG in lower/higher Mg groups. MPG: mean aortic valve pressure gradient *p* value <0.001 (repeated measures mixed-effects model) and data given as mean ± SD. Serum Magnesium levels: Lower Mg group, 1.9–2.3 mg/dL; Higher Mg group, 2.4–3.0 mg/dL. (b) Change of EOAi in lower/higher Mg groups. EOAi: Effective Orifice Area index *p* value <0.001 (repeated measures mixed-effects model) and data given as mean ± SD.

Next, postoperative changes of MPG were compared between the 2 subgroups categorized by median values of preoperative sMgs (Figure 1(a)). In lower Mg group (Mg: 1.9–2.3 mg/dL), MPG significantly increased over time (*p* < 0.001, repeated measures mixed-effects model). We also found that EOAi was significantly decreased after TAVI (*p* < 0.001, repeated measures mixed-effects model) in lower Mg group as compared in higher Mg group (Mg: 2.4–3.0 mg/dL, Figure 1(b)).

Of the 24 patients in this study, 3 underwent reoperation (TAV-in-TAV), and indeed, the preoperative sMgs in these 3 patients (reoperation group) were in the range of 1.9–2.1 mg/dL, predominantly lower compared to the other group (Table 4, *p* = 0.04, Wilcoxon Signed-Rank Test). In addition, 6 of the 24 patients were classified as moderate/severe stenosis and showed significantly lower sMgs (Table 5, *p* = 0.049, Wilcoxon Signed-Rank Test).

**Table 4.** Baseline levels of serum magnesium in non-reoperation and reoperation group.

	Non-reoperation (*n* = 21)	Reoperation (*n* = 3)	*p* Value
	Median [IQR]	Median [IQR]	
Magnesium, mg/dL	2.4 [2.2–2.7]	2.1 [1.9–2.1]	0.04

**Table 5.** Baseline levels of serum magnesium in Non/Mild stenosis and Moderate/Severe stenosis group.

	Non/mild stenosis (*n* = 18)	Moderate/ severe stenosis (*n* = 6)	*p* Value
	Median [IQR]	Median [IQR]	
Magnesium, mg/dL	2.5 [2.3–2.9]	2.2 [2.1–2.2]	0.049

MPG increased not immediately after but one week (*p* = 0.01) after surgery (Table 6). All three cases showed valve calcification before reoperation, indicating that SVD was due to severe stenosis caused by calcification. The median follow-up period after TAV in TAV was 4.1 years [0.79–5.1] without restenosis.

**Table 6.** Comparison of MPG between non-reoperation and reoperation groups over time.

	Overall (*n* = 24)	Non-reoperation (*n* = 21)	Reoperation (*n* = 3)	*p* Value
	Median [IQR]	Median [IQR]	Median [IQR]	
MPG 1D, mm Hg	9 [5–10]	8 [5–9]	14.5 [9–20]	0.2
MPG 1W, mm Hg	9 [7–12.5]	8 [7–10]	16 [13–20]	0.01
MPG 1M, mm Hg	9 [8–14]	9 [7.5–12.5]	12 [9–15]	0.5
MPG 6M, mm Hg	10 [9–12]	10 [8–11]	17 [14–20]	0.01

MPG 1D/1W/1M/6M: mean aortic pressure gradients at 1 day/1 week/1 month/6 months after operation.

## Discussion

Our results showed that preoperative sMgs were associated with changes in MPG, an index of SVD due to valve stenosis after TAVI in HD patients. Indeed, the three patients who presented SVD were all due to calcific valve stenosis and showed low levels of sMg. Furthermore, analysis by linear mixed-effects models for repeated measures revealed that preoperative sMgs negatively correlated with MPG. Taken together, preoperative hypomagnesemia could be a valuable predictor for bioprosthetic valve stenosis after TAVI.

Perturbated bone-mineral metabolism is commonly observed in patients with chronic kidney disease and is closely related to vascular calcification and cardiovascular diseases. Generally, vascular and valvular calcification is more prominent in HD patients than in non-HD patients. Active inducers of calcification including secondary hyperparathyroidism, hyperphosphatemia, hypercalcaemia, oxidative stress and uremic toxins could be responsible for both vascular and valvular calcification [15]. Mg is the fourth most abundant cation dominantly in the bone and also work as an inhibitor of calcification via preventing phosphate-induced osteogenic differentiation. Magnesium also was reported to suppress vascular calcification by activating TRPM7, inducing expression of anti-calcification proteins (osteopontin, BMP-7, matrix Gla protein), and inhibiting Wnt/β catenin signaling [16–18]. In clinical studies, some report that Mg can prevent coronary artery calcification in patients with chronic renal failure [19,20], that magnesium carbonate administration reduces the severity of ectopic calcification [21,22]. Some cohort studies have reported associations between interventions on sMgs and cardiovascular outcomes and aortic valve calcification [15,23].

In HD patients, sMgs generally range between normal to low before HD [24,25]. Considering Mg concentration of most dialysates in Japan is set as 1.0 mEq/L (1.21 mg/dl), sMgs of patients in this study might be dependent on mineral metabolic status as well as of dietary pattern. Low pre-dialysis sMg increase cardiovascular risks such as high pulse pressure, high left ventricular mass index and vascular calcification, and what is more, increase mortality in HD patients [26]. Unlike other minerals, the importance of magnesium in preventing cardiovascular events such as calcification of blood vessels or aortic valves has been less focused.

Furthermore, calcification of aortic valves may differ slightly from that of blood vessels, and there is still much that is unknown about the mechanism. There are several reports on the mechanism of aortic valve calcification. It is thought that osteoblast-like cells appear in valves as well as in blood vessels, which are involved in tissue calcification [12,27,28]. Endothelial cells are present on the surface of the valve, interstitial cells in layers between them, alternating with collagen, glycosaminoglycans and elastin. The interstitial cells play an important role in the valve, synthesizing extracellular matrix and transforming into cardiac fibroblasts and osteoblasts [29,30]. In addition to abnormalities in mineral metabolism, valve calcification is thought to be related to oxidized LDL cholesterol, hypertension, biomechanical abnormalities, oxidative stress, inflammation, and the renin-angiotensin system [29,31,32].

It is previously reported that serum IP was positively correlated with the prevalence and incidence of aortic valve calcification, and Mg were inversely correlated, suggesting that these serum micronutrients may be potential candidates for risk prediction or prevention of aortic valve calcification [33]. In our study, the bone-mineral metabolism biomarkers such as Ca, IP and iPTH had smaller effect on the increase of MPG in the target population of this study. Since the control of these parameters in HD patients depends on dialysis management and oral medication, it is possible that they were relatively well controlled in the dialysis clinic. As compared to these fluctuating parameters, Mg status is less likely to change in the short term, therefore baseline hypomagnesemia may be appropriate as predictors of dysfunction of bioprosthetic valve after TAVI. Although the timing of measurement varies, the mean Mg levels after TAVI showed a relatively high correlation with the mean sMgs before TAVI (*R*^2^ = 0.54, data not shown) and no patient received Mg supplementation at the time of operation. Taken together, it is expected to conduct intervention studies to validate the evidence of Mg treatment in the future.

New approach to prevent reoperation by improving surgical devices has recently been attempted, such as the use of newer generation balloon-expandable valve (i.e., Acurateneo, Sapien 3, etc.) which is reported to improve the mid- to long-term prognosis [34,35]. Not only from the surgical point of view but also from dialysis management, it is important to investigate further for identifying risks in debasing valve durability in order to improve long term prognosis after TAVI in HD patients.

MPG was mainly focused on as criteria for valve stenosis in this study, but it may be difficult to interpret echocardiographic findings in dialysis patients, due to large changes in blood volume [36]. We evaluated postoperative MPG and EOAi, and found significant increase of MPG and decrease of EOAi in lower sMg group. However, MPG, but not EOAi was related to preoperative sMgs in linear mixed-effects models. This discrepancy is probably due to relatively low intercorrelation between postoperative MPG and EOAi (*R*^2^= 0.32, data not shown).

There are some limitations in this study. This is a single-centre observational study with a quite small number of cases and limited statistical power. The evaluation was based on data from a preoperative dialysis clinic, and there may be differences in the measurement system. In HD patients, Mg tends to be withdrawn from the body through the dialyzer, resulting in low Mg status, and thus the factors that contribute to elevated sMg are largely dependant on daily life (especially diet). Management of sMg is controversial because of the limited range of increase with supplementary therapy [20]. Mg status in non-HD patients remains unclear, and whether these findings are unique to HD patients need to be clarified in the future.

In conclusion, we studied preoperative clinical factors and postoperative course of bioprosthetic valves in HD patients who underwent TAVI. We found that preoperative hypomagnesemia was associated with the increase of MPG after TAVI among bone-mineral metabolism markers. Although this is a single-centre study with a small size and need to be analyzed in larger number of patients, our study suggests that hypomagnesemia could predict post-TAVI valve dysfunction in HD patients.
